# Neuroprotective Effects and Metabolomics Study of Protopanaxatriol (PPT) on Cerebral Ischemia/Reperfusion Injury In Vitro and In Vivo

**DOI:** 10.3390/ijms24021789

**Published:** 2023-01-16

**Authors:** Fulin Wu, Sihan Lai, Dongxing Fu, Juntong Liu, Cuizhu Wang, Hao Feng, Jinping Liu, Zhuo Li, Pingya Li

**Affiliations:** 1 School of Pharmaceutical Sciences, Jilin University, Changchun 130021, China; 2 College of Basic Medicine Sciences, Jilin University, Changchun 130021, China

**Keywords:** protopanaxatriol, cerebral ischemia/reperfusion, neuroprotective, metabolomics

## Abstract

Stroke, one of the leading causes of disability and death worldwide, is a severe neurological disease that threatens human life. Protopanaxatriol (PPT), panaxatriol-type saponin aglycone, is a rare saponin that exists in *Panax ginseng* and *Panax Noto-ginseng*. In this study, we established an oxygen-glucose deprivation (OGD)-PC12 cell model and middle cerebral artery occlusion/reperfusion (MCAO/R) model to evaluate the neuroprotective effects of PPT in vitro and in vivo. In addition, metabolomics analysis was performed on rat plasma and brain tissue samples to find relevant biomarkers and metabolic pathways. The results showed that PPT could significantly regulate the levels of LDH, MDA, SOD, TNF-α and IL-6 factors in OGD-PC12 cells in vitro. PPT can reduce the neurological deficit score and infarct volume of brain tissue in rats, restore the integrity of the blood-brain barrier, reduce pathological damage, and regulate TNF-α, IL-1β, IL-6, MDA, and SOD factors. In addition, the results of metabolomics found that PPT can regulate 19 biomarkers involving five metabolic pathways, including amino acid metabolism, arachidonic acid metabolism, sphingolipid metabolism, and glycerophospholipid metabolism. Thus, it could be inferred that PPT might serve as a novel natural agent for MCAO/R treatment.

## 1. Introduction

Ischemic stroke is a serious neurological disease that threatens human life, it is caused by arterial stenosis or focal occlusion in the brain [1]. The key point of clinical treatment of ischemic stroke is the early recovery of blood reperfusion [2]. Brain tissue is very sensitive to ischemia and hypoxia. The blood reperfusion into ischemic brain tissue may cause severe inflammation, oxidative stress injury, and neuron and cell death due to calcium overload, free radicals’ occurrence, and other factors [3,4]. Clarifying the mechanism of cerebral ischemia-reperfusion (CIR) injury is currently a hot topic in science [5]. The two most crucial therapy strategies for ischemic stroke currently are thrombolysis and neuroprotection. Nevertheless, the therapeutic window for thrombolytic medications and neuroprotective therapies is narrow, and their efficacy is insufficient [6]. A neuroprotective agent with a significant clinical impact, adequate safety, and few side effects are therefore urgently needed for the treatment of ischemic stroke.

Protopanaxatriol (PPT), panaxatriol-type saponin aglycone, is a rare saponin that exists in Panax ginseng and Panax Noto-ginseng; two traditional Chinese medicines which have been used clinically for thousands of years in China and are now widely used around the world [7]. It has been reported that ginsenosides can be hydrolyzed in the gastrointestinal tract to form their more easily absorbed metabolites and show stronger activity than their original saponins [8,9,10]. Many studies have shown that ginsenoside Rg1 has a good anti-cerebral ischemic injury effect [11,12]. Protopanaxatriol, a metabolite of ginsenoside Rg1, has similar and more potent effects in improving memory and hippocampal excitability, suggesting that the role of the sugar moiety in biological activity is not as necessary as traditionally thought [13]. At present, the effect and mechanism of protopanaxatriol in ischemic stroke disease have not been reported.

Metabolomics is a technique for analyzing the entire endogenous metabolites in living systems, which can describe the dynamic changes of metabolites triggered by external perturbations in the whole biological system. It has been widely employed in the development of biomarkers, the evaluation of toxicity, and the evaluation of medication efficacy [14,15,16]. Currently, liquid chromatography-mass spectrometry (LC-MS), gas chromatography-mass spectrometry (GC-MS), and nuclear magnetic resonance (NMR) are the most commonly used techniques in screening targets in metabolomics. LC-MS is the most widely used and promising metabolomics technology because it is high-resolution and high sensitivity enough to be used in complex systems such as plasma and tissue [17,18]. It can elucidate and reflect the pathogenesis of complex diseases in general and can effectively help diagnose diseases and evaluate prognosis.

In the presented work, firstly, the oxygen-glucose deprivation (OGD)-PC12 cell model was established to detect the anti-inflammatory and antioxidant activity factors of PPT in vitro. Then, we evaluated the protective effect of PPT against cerebral ischemia/reperfusion (CIR) injury and investigated the underlying mechanism through metabolomics. These results may provide evidence to support PPT as a treatment for ischemic stroke.

## 2. Results

### 2.1. Effect of PPT on OGD-PC12 Cell

#### 2.1.1. Cell Viability

**Figure 1:**
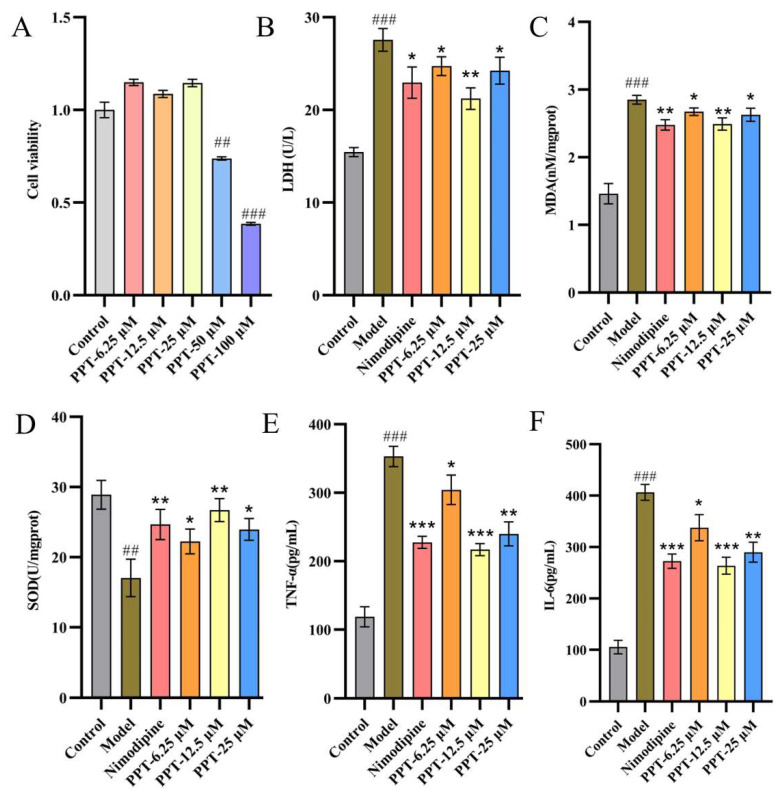
(**A**) The impact of PPT on the viability of PC12 cells. (**B**–**F**) Effects of PPT on LDH, MDA, SOD, TNF-α, and IL-6 levels in OGD-P12 Cells. (*n* = 6); Compared with the control group, ## *p* < 0.01, ### *p* < 0.001; compared with the model group, * *p* < 0.05, ** *p* < 0.01, *** *p* < 0.001.

The result of the PC12 cell viability assay is shown in Figure 1A. Compared with the control group, low dosages (6.25 μM, 12.5 μM, 25 μM) of PPT had no significant effect on the survival rate of cells, but 50 μM and 100 μM of PPT could significantly inhibit the cell viability (*p* < 0.01, *p* < 0.001). Therefore, 6.25 μM, 12.5 μM, and 25 μM were chosen as the dosage of PPT to investigate the anti-inflammatory and antioxidant activity.

#### 2.1.2. Anti-Inflammatory and Antioxidant Activity

As shown in Figure 1B, the OGD model was successfully induced, as evidenced by the model group’s considerably higher levels of LDH, MDA, SOD, TNF-, and IL-6 when compared to the control group (*p* < 0.001). The levels of LDH, MDA, TNF-, and IL-6 may be considerably decreased to varying degrees following the administration of PPT or Nimodipine, as compared to the model group (*p* < 0.05, *p* < 0.01, *p* < 0.001). Notably, PPT had antioxidant and anti-inflammatory effects that were comparable to those of nimodipine (*p* < 0.05). According to the preceding study’s findings, PPT effectively protected OGD-PC12 in vitro.

### 2.2. Effect of PPT on CIR Injury Rats

#### 2.2.1. Evaluation of Neurological Deficit Function Score and Infarct Volume

**Figure 2:**
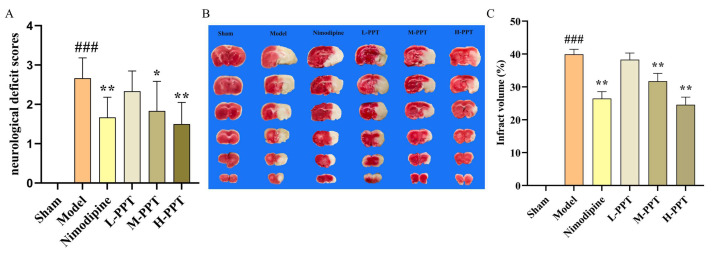
Effect of PPT improves the ischemia-reperfusion injury in rats. The results of neurological deficit score in different groups (**A**) (*n* = 6); the TTC staining and infraction rate of brain tissue in different dose groups (**B**,**C**) (*n* = 3). ^###^ *p* < 0.001 vs. control. * *p* < 0.05, ** *p* < 0.01 vs. model group.

After 2 h ischemia and 22 h reperfusion, the model was established, all rats survived. The neuroprotective impact of PPT was determined through evaluation of the neurological deficit using Longa’s method among the different groups. As shown in Figure 2A, the rats in sham group did not have any neurological defect and had a score of zero, while the rats in the model group showed significantly higher scores that in the sham group. Interestingly, the neurological deficit score in the PPT and nimodipine-treated groups was lower than those of the model group. Pre-treatment with H-PPT, M-PPT, and nimodipine resulted in a significant improvement in the neurological deficit compared with the model group (*p* < 0.05).

The TTC-staining infarct volume of each group was shown in Figure 2B,C, no infarct area was observed in the brain tissue of the rats in the sham group. The results demonstrated that the infarct volume in all the treated groups was lower than those of the Model group. Specifically, the infarct volumes in the groups that were treated with H-PPT and M-PPT or nimodipine were significantly lower than that of the model group (*p* < 0.05).

These results indicate that pretreatment with PPT attenuated cerebral damage following ischemia-reperfusion injury.

#### 2.2.2. Blood-Brain Barrier Integrity Measurement

**Figure 3:**
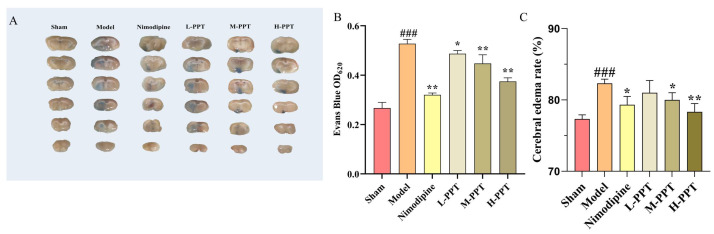
Effect of PPT on blood-brain barrier disruption (*n* = 3). The staining and content determination of Evans blue in different groups (**A**,**B**). The change in cerebral edema rate in different groups (**C**). ### *p* < 0.001 vs. sham. * *p* < 0.05, ** *p* < 0.01 vs. model group.

We assessed the permeability of the blood-brain barrier using the Evans blue staining method. The results are shown in Figure 3A,B, we found that the content of Evans blue in the model group was significantly higher than that in the sham group, indicating cerebral ischemia and significant damage to the blood-brain barrier. Compared with the model group, the content of Evans blue in the PPT pretreatment group was significantly lower. Especially, H-PPT, M-PPT, and nimodipine groups had significantly decreased content. Cerebral ischemia injury can lead to cerebral edema, as shown in Figure 3C, the brain tissue of the model group rats contains water content significantly higher compared with the Sham group. The brain tissue water content decreased by pretreatment with PPT and H-PPT, M-PPT and nimodipine resulting in a significant improvement. These results indicate that PPT can improve the blood-brain barrier damage caused by cerebral ischemia-reperfusion to a certain extent.

#### 2.2.3. Pathological Evaluation of Brain Tissue

**Figure 4:**
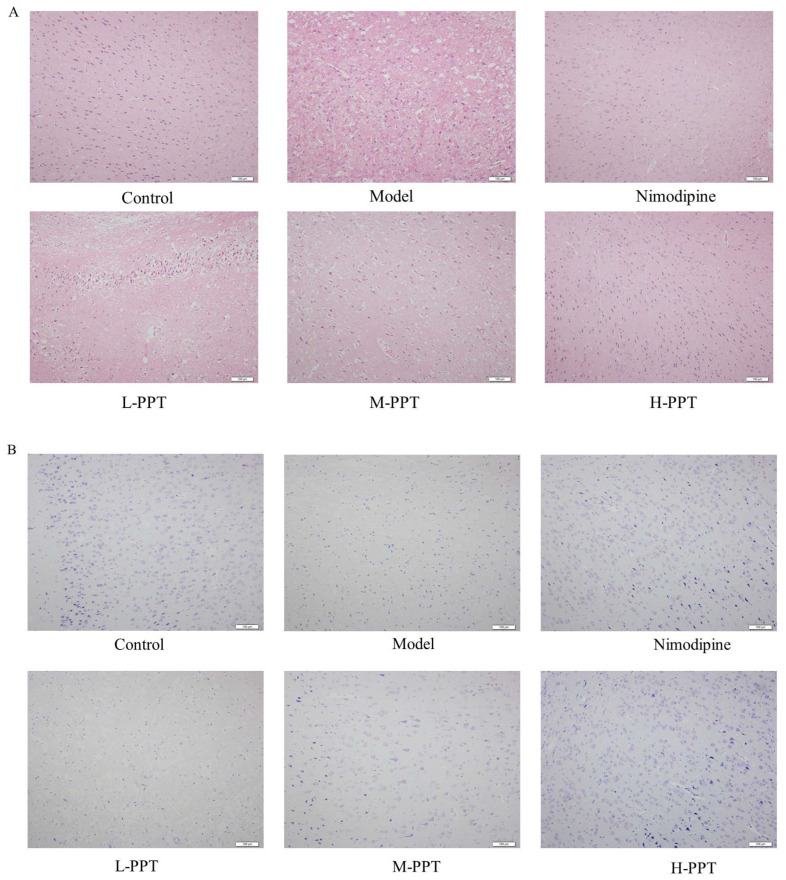
Detection of brain tissue injury in different groups. The representative histopathological changes in different groups by HE staining and Nissl staining method (**A**,**B**), Scale bar = 100 µm (*n* = 3).

As shown in Figure 4A, hematoxylin and eosin (H&E) staining of the cerebral cortices of the sham group demonstrated an intact cortical structure with intact cell nuclei and homogeneous cytoplasm, compared with the control group, there are large number of vacuoles and nuclear shrinkage in the cortex and striatum of the model group, with irregular shapes and partial nuclear chromatin dense and dense shrinkage, partial nuclear pyknosis occurs, and a lot of damage occurs. Pretreatment with H-PPT, M-PPT, and nimodipine successfully reversed the CIR-induced histopathological changes. Whereas, pretreatment with L-PPT did not have obvious neuroprotective effects.

Through the results of H&E staining, we found that PPT can reduce the damage of brain tissue, then we simultaneously combined it with the method of Nissl staining, and the effect of PPT in cerebral ischemia-reperfusion injury was further verified. As shown in Figure 4B, the results showed that compared with the control group, the expression of Nissl bodies in neurons in the Model group was significantly decreased, and the intercellular space of neurons was increased, indicating that the degree of neuronal damage was higher. However, pretreatment with H-PPT, M-PPT, and nimodipine significantly increased Nissl bodies in neuron numbers indicating that neuronal damage was further alleviated.

#### 2.2.4. Results of Biochemical Parameters

**Figure 5:**
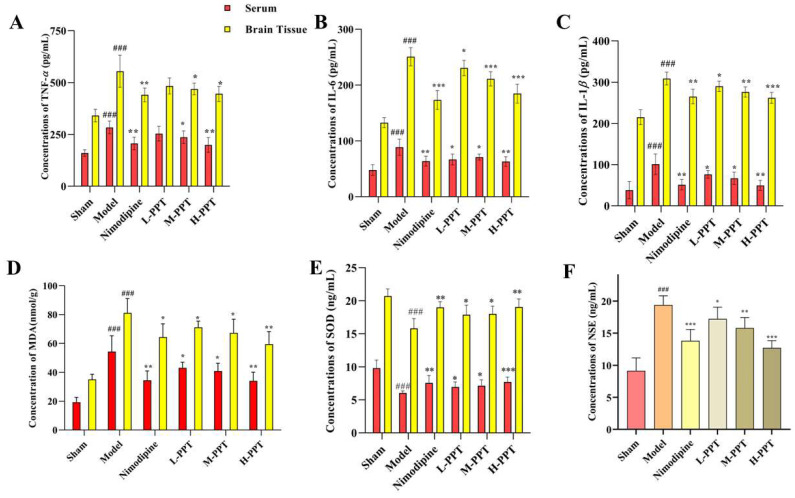
The result of the Elisa analysis. TNF-α, IL-6, IL-1β, MDA, and SOD of the serum and brain in the different groups (**A**–**E**); NSE of serum in the different groups (**F**). ### *p* < 0.001 vs. sham. * *p* < 0.05, ** *p* < 0.01, *** *p* < 0.001 vs. model group.

In order to learn more about the inflammatory and oxidative effects of PPT on cerebral ischemia/reperfusion injury, cytokines were found in the rats’ serum and brains. Our findings (Figure 5) demonstrated that the model group’s serum and brain tissue levels of TNF-, IL-6, IL-1, and MDA were considerably greater than those of the sham group (*p* < 0.001), and the model group significantly decreased the levels of SOD (*p* < 0.001), showing that the model group experienced a strong inflammatory and oxidation response.

PPT significantly reduces the levels of TNF-α, IL-6, IL-1β, and MDA (Figure 5A–D) and significantly increased the levels of SOD in serum and brain tissues (Figure 5E), except low doses with PPT, has not significantly different from a model in TNF-α, others dose group with PPT have notably affected (*p* < 0.05), and the activity of PPT-H group is the same as that of nimodipine group. The results show that PPT has a significant anti-inflammatory and anti-oxidative effect. We also tested nerve damage-specific indicators NSE (Figure 5F), and the results expressed PPT can reduce the content of NSE in the serum (*p* < 0.05), thereby protecting ischemia/reperfusion injury.

### 2.3. Results of the Serum and Brain Tissues Metabolomics

#### 2.3.1. Validation of UPLC-QTOF-MS

The *m*/*z*-retention time (RT, min) pairs chosen for the system stability test in the positive electrospray ionization (ESI^+^) mode included: 146.0812–0.76; 756.8344–6.53; 874.4466–10.91; 362.3269–13.16; 603.4112–17.93; 303.2328–18.27; 637.3029–23.33; 775.5743–28.65, and in negative electrospray ionization (ESI^−^) mode, the m/z-RT pairs were as follows:103.0458–0.87, 187.0119–4.38, 391.2872–15.45, 557.3184–18.11, 568.3591–20.19, 337.2090–22.31, 327.2365–22.56, 235.9308–29.42.

The system stability, precision, repeatability, and sample stability tests’ relative standard deviations (RSDs) of the peak intensities (PI) and (RT) were provided in (Supplementary Table S1).

The UPLC-QTOF-MS method was successful in terms of precision, reproducibility, and stability, according to the above validation.

The established method was employed to locate the serum and brain samples. Additionally, representative base peak intensity (BPI) chromatograms for each group were shown in (Supplementary Figure S1).

#### 2.3.2. Multivariate Statistical Analyses

**Figure 6:**
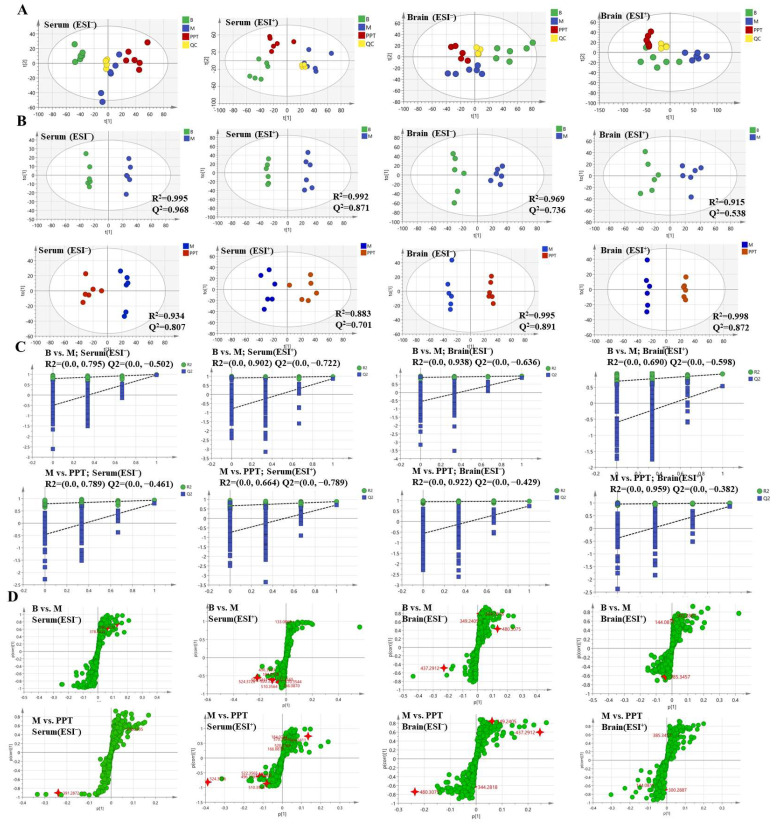
The metabolic profile of serum and brain are shown in PCA score (**A**), OPLS-DA score (**B**), permutations test plots (**C**), and OPLS-DA S-plots (**D**). “B” represents sham group; “M” represents model group.

Principal component analysis (PCA) was performed for serum and brain metabolic profiles of mice for each group (Figure 6A). Compared to the experimental samples, the QC samples were clustered and had good repeatability. The study of the PCA data showed that the model group, the sham group, and the H-PPT group could all be easily separated from one another in the PCA score plot in the positive and negative ion modes. This suggests that both the treatment groups and the model group experienced biochemical interference. Despite this, the H-PPT treatment group inclined toward the control group whereas the model group was significantly separated from each group and each group was heavily clustered, demonstrating that the rats’ health had significantly improved following H-PPT therapy.

In addition, the model OPLS-DA was constructed to improve the discriminant analysis ability of the model and further determine the different components between the model group and the H-PPT treatment group. The groups were divided because the categorization effect was significant (Figure 6B), indicating that the metabolite characteristics of the rats were significantly improved by the H-PPT treatment group.

When Q2 is greater than 0.5, the OPLS-DA model’s predictive validity is generally strong. The original model is valid and there is no overfitting, as shown by the permutation plots, which show that all blue Q2 points are lower than the original right points (Figure 6C). The covariance and correlation between the metabolites and the modeled class are assessed using the S-plot. It was created to identify the potential metabolites (Figure 6D). The further away from the origin, the more significant the points in the S-plots contribute to the clustering of the model group and the H-PPT treatment groups.

#### 2.3.3. Screening and Identification of Metabolites

**Figure 7:**
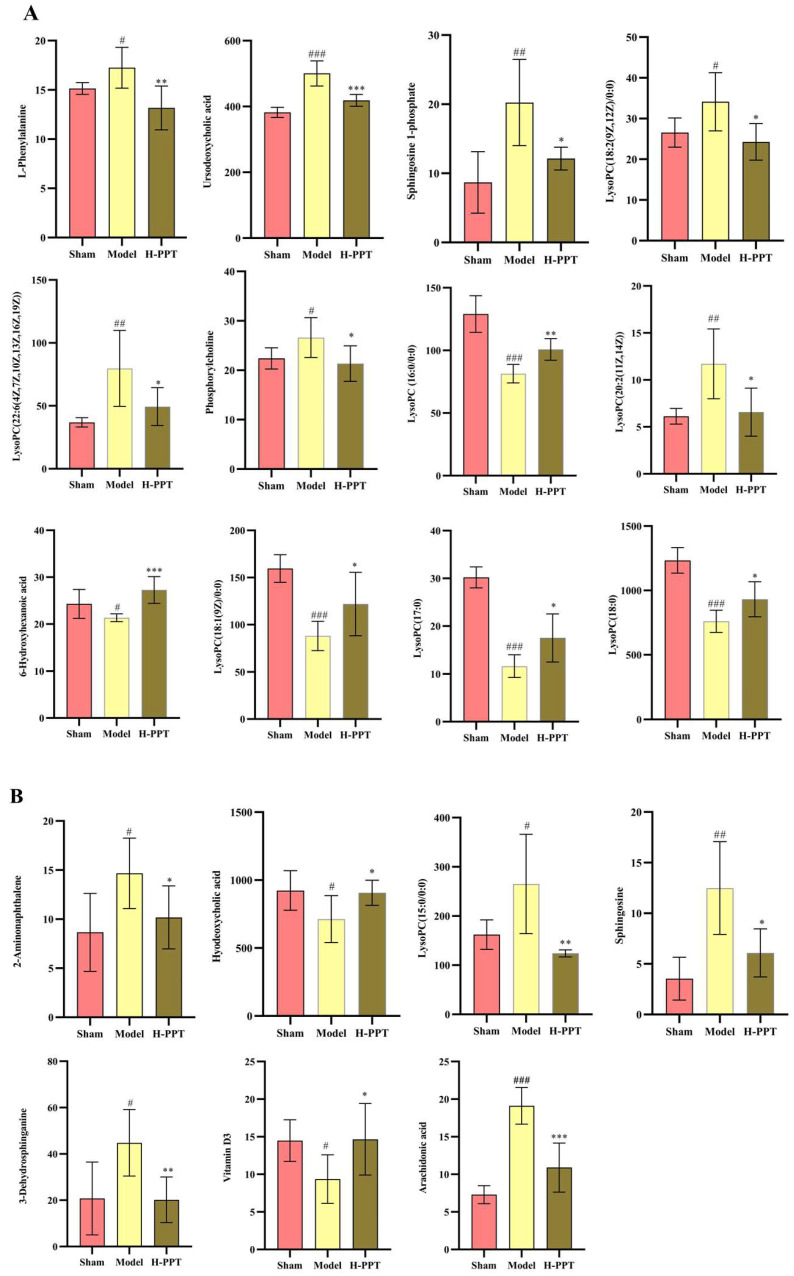
Each biomarker’s concentration in the sham, model, and H-PPT groups’ serum (**A**) and brain (**B**). Data are presented as means ± SD (*n* = 6). Compared with control group, # *p* < 0.05, ## *p* < 0.01, ### *p* < 0.001; compared with model group, (* *p* < 0.05, ** *p* < 0.01, *** *p* < 0.001).

The putative biomarkers were selected from construct S-plots. The metabolites could only be taken into consideration as potential biomarkers when the value of variable importance in the projection (VIP), the combination VIP > 1 in the S-plot diagram, and the *p*-value < 0.05. The MS/MS fragments and online databases were used to identify endogenous metabolites. Based on the procedures outlined above and metabolite comparisons between the model group and the H-PPT treatment group, 19 different biomarkers were identified (Table 1). They are thought to be endogenous MCAO/R biomarkers and their levels were strongly modulated by H-PPT. Figure 7 shows the concentrations of each biomarker in the sham, model, and H-PPT groups’ serum and brain.

**Figure 8:**
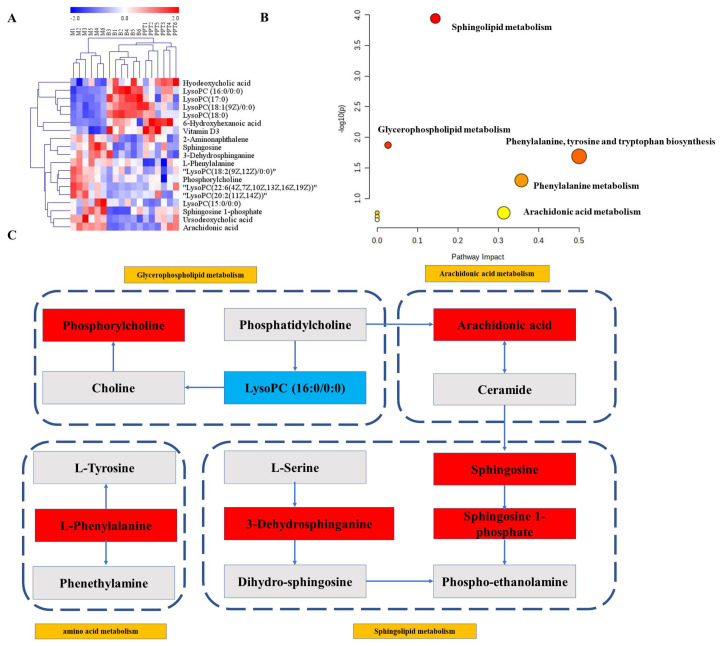
(**A**) The heat map of biomarkers. (**B**) Effects of PPT on main metabolic pathways in UC. (**C**) The metabolic network between main biomarkers and metabolisms.

Hierarchical clustering heat maps were then constructed using the identified metabolites (Figure 8A). In this heat map, the color from red to blue indicated decreasing abundance of biomarkers. To explore the potential metabolic pathways in which the level of these biomarkers was significantly regulated by H-PPT, 19 endogenous biomarkers were imported into Metaboanalyst to perform pathway enrichment analysis (Supplementary Table S2). The analysis results showed that five metabolic pathways were potential target metabolic pathways of H-PPT in the treatment of UC in mice (Figure 8B). In addition, we mapped relative metabolic pathways concerning the KEGG database (Figure 8C). The network showed that the H-PPT treatment increased the blue metabolites and downregulated the red ones. This indicates that H-PPT can regulate the disruption of these metabolic pathways.

## 3. Discussion

Nowadays, cerebral ischemic injuries still carry a high mortality rate globally. They can lead to a complex pathology of inflammatory injury, increased oxidative damage, energy metabolism dysfunction and even excitotoxicity, all of which impact the severity, and condition’s prognosis [19]. In particular, during a certain period after cerebral ischemia-reperfusion, the brain’s unrecoverable function, and more severe brain dysfunction known as cerebral ischemia-reperfusion injury [20]. However, the pathogenesis of cerebral ischemic diseases remains unclear. Researchers are trying their best to elucidate the mechanism and find effective new drugs for treatment, but results are far from satisfactory. There is a new research direction to discover active ingredients against cerebral ischemic injury from natural products. In recent years, many active compounds such as Puerarin [21], Curcumin [22,23], and Salvianolic acid B [24] have been discovered.

To the extent that we are aware, this study was the first to study the effect of PPT against cerebral ischemia-reperfusion injury. Our findings demonstrate that PPT strongly regulates the levels of LDH, MDA, SOD, TNF-α, and IL-6 factors as well as considerably protects OGD-PC12 cells in vitro. Pre-treatment with PPT considerably reduced cerebral edema, lowered infarct volume, and neurological impairment in vivo. It also improved blood-brain barrier function. Additionally, it can control the amounts of oxidative factors and decrease inflammatory factors in rats with CIR. According to the findings of the pathological assessment, PPT can dramatically lessen neuronal damage, enhance cell shape, and enhance the number of Nissl bodies.

The central nervous system could be harmed by CIR through oxidative stress, neuroinflammation, and other processes, according to numerous research [25]. This can lead to various sequelae and problems, including cognitive decline [26] and gastrointestinal bleeding [27]. An imbalance between oxidation and antioxidants results in oxidative stress [28]. SOD, MDA, and LDH factors were used to measure and monitor oxidative stress [29]. In addition, inflammatory cytokines are crucial inflammatory response mediators in disorders of the central nervous system [30]. Cerebral ischemia induces the expression of adhesion molecules and the production of large amounts of proinflammatory cytokines in endothelial cells and leukocytes, understanding how inflammation affects the CIR may offer a new approach to treating it. TNF-α, IL-6, and IL-1β were involved in mediating of inflammatory cytokines during cerebral CIR [31]. Our research shows that PPT can improve these inflammatory and oxidative factors, indicating that PPT can resist CIR injury by anti-oxidative stress and anti-inflammation, and achieve neuroprotective effects.

Since the etiology and pathophysiology of CIR are not fully understood, a new technique, metabolomics, has been increasingly used to explore the mechanisms of CIR [32]. The presented metabolomic investigation showed that H-PPT therapy clearly interfered with the 19 biomarkers implicated in five metabolisms. Therefore, we assumed that the more potent pharmacological effects of H-PPT must be connected to the five metabolites’ pathways.

In amino acid metabolism, the aromatic amino acid called L-phenylalanine is crucial for producing other amino acids [33]. When present at very high levels, phenylalanine acts as a neurotoxin and can damage nerve cells and tissues [34]. According to studies, brain ischemia damage can result in a rise in the phenylalanine [35]. According to our findings, phenylalanine levels in the model group were considerably higher than in the sham one, which is in line with other studies [36]. We hypothesize that brain injury alters the metabolism of phenylalanine, leading to the buildup of phenylalanine and brain cell destruction. Giving H-PPT therapy allows us drastically reverse this condition.

The pathological response to brain injury causes neuroinflammation, which produces a flood of pro-inflammatory chemicals and damages neurons [37]. Arachidonic acid metabolism is closely related to inflammation. Arachidonic acid enzymatically converts to bioactive prostanoids that play a central role in the acute inflammatory cascade [38]. We found that arachidonic acid content significantly increases in the cerebral ischemia model, which is consistent with previous literature reports [39]. After treatment with H-PPT, the content of arachidonic acid can be reduced, this may be the cause of anti-inflammation.

Studies have shown that lipids may be closely related to the pathological development of brain injury [40]. Lyso-phospholipid, an intermediate product of phospholipid metabolism, was believed to be closely related to oxidative stress injury [41]. Phospholipases can hydrolyze phospholipids to lysophosphatidylcholine (LPC), and lysophosphatidylethanolamine (LPE), resulting in the production of arachidonic acid [42]. In our study, we identified nine metabolites in glycerophospholipid metabolism. Among them, eight are lysophospholipid metabolites, and one is phosphorylcholine. The content of LysoPC(18:2(9Z,12Z)/0:0), LysoPC(20:2(11Z,14Z)), LysoPC(22:6(4Z,7Z,10Z,13Z,16Z,19Z)), LysoPC(15:0/0:0) and phosphorylcholine were significantly increased in the IR model group, while the lever of LysoPC (16:0/0:0), LysoPC(18:1(9Z)/0:0), LysoPC(17:0) and LysoPC(18:0) were decreased. After giving H-PPT interference, the content of metabolites can be significantly adjusted, tending to the sham group, implying that H-PPT can play an anti-ischemic effect by regulating glycerophospholipid metabolism.

Disorders of the sphingolipid metabolism are closely related to a variety of neurodegenerative diseases, which may lead to their complex pathogenesis [43]. Sphingosine is a bioactive substance that has a role in cell differentiation, autophagy, transcription, and apoptosis, all of which are connected to inflammatory disorders [44]. Sphingosine 1-phosphate is a bioactive sphingolipid metabolite that plays a regulatory role in neurotransmitter release, synaptic transmission, and neuroinflammation [45]. In the CIR model group, we found that the content of metabolites of Sphingosine, Sphingosine 1-phosphate, and 3-Dehydrosphinganine increased significantly, indicating that sphingolipid metabolism was affected, which is consistent with a previous report [46], and the accumulation of sphingolipid metabolites led to brain damage. After treatment with H-PPT, the levels of the three metabolites all decreased, tending to the sham group.

## 4. Materials and Methods

### 4.1. Chemicals and Reagents

Protopanaxatriol (PPT) was obtained from the Natural Medicine Research Center of Jilin University (batch number 211215). 2,3,5-Triphenyl-2H-tetrazolium chloride (TTC) was purchased from Solarbio Science & Technology Co., Ltd. (Beijing, China). Nimodipine (NMDP) tablets were purchased from the Yabao Pharmaceutical Group Co., Ltd. (Shanxi, China); L-Phenylalanine, Vitamin D3 and Arachidonic acid were obtained from Sichuan Weikeqi Biotechnology Co., Ltd. (Chengdu, China). Neuron-specific enolase (NSE) was acquired from Shanghai Zhuocai Biotechnology Co., Ltd. (Shanghai, China); Lactate dehydrogenase (LDH) assay kit, Superoxide dismutase (SOD) and Malondialdehyde (MDA) assay kits were purchased from the Nanjing Jiancheng Bioengineering Institute (Nanjing, China), Interleukin-1β (1L-1β), interleukin-6 (IL-6), and tumor necrosis factor-α (TNF-α) enzyme-linked immunosorbent assay kits were purchased from Hangzhou Lianke Biotechnology Co, Ltd. (Hangzhou, China). Nissl Staining Solution (Cresyl Violet) was purchased from Solarbio Science & Technology Co., Ltd. (Beijing, China); 4% paraformaldehyde solution was purchased from Biyuntian Co, Ltd. (Shanghai, China).

Cell-grade DMSO and Cell Counting Kit-8 (CCK-8) were obtained from Solarbio Science & Technology Co., Ltd. (Beijing, China). DMEM high glucose and sugar-free medium (DMEM) was obtained from HyClone Biochemical Co., Ltd. (Beijing, China). Fetal bovine serum (FBS) was obtained from Thermofisher Scientific Co., Ltd. (Beijing, China). Penicillin and streptomycin were obtained from Shanghai Yubo Biotechnology Co., Ltd. (Shanghai, China). Phosphate buffer saline (PBS) was obtained from Nanjing Shenghang Biotechnology Co., Ltd. (Nanjing, China).

UPLC-grade acetonitrile and methanol were acquired from Thermofisher Scientific (Shanghai, China). Deionized water was obtained from A.S. Watson Bunch Ltd. (Hong Kong, China). All other chemical solvents were of analytical grade.

### 4.2. Cell Culture and Passaging

The Cell Bank of the Chinese Academy of Sciences provided the differentiated PC12 cells (Shanghai, China). The cells were maintained at DMEM medium supplemented with 10% FBS with 1% of two types antibiotics (penicillin and streptomycin). The cultures were maintained in 37 °C and 5% CO_2_. The media was changed once daily. When the cells covered 70–80% of the bottom of the bottle, the medium in the cell culture dish was discarded. The cultures were washed twice with PBS, 1 mL of fresh medium was added and the cells were gently blown with fresh medium, then we waited until the cells were completely detached, and centrifuged the cell suspension at 1000 rpm/min for 5 min. After discarding the supernatant, an appropriate amount of fresh medium was added to resuspend the cells, and they were transferred to cell culture dishes according to the cell density.

### 4.3. Oxygen-Glucose Deprivation/Reperfusion (OGD/R) Model

PC12 cells were cultured in serum-free DMEM medium without glucose to simulate ischemia and to simulate hypoxia cells were cultured in a three-gas incubator filled with 1% O_2_, 5% CO_2_, and 94% N_2_ for 12 h, then discarded the old medium, added complete medium and placed it in a 5% CO_2_ incubator at 37 °C for 4 h to simulate reperfusion.

### 4.4. Cell Viability

On a 96-well plate, PC12 cells (5 × 10^4^ cells/well) were planted, cultivated to a density of 70–80%, and the medium was then discarded. Six groups were created from the cells. Cells were cultivated for 24 h in PPT groups (6.25, 12.5, 25, 50, and 100 µM) and the control group. The cells were subsequently given CCK-8, and they were left to incubate for an additional 2 h. Additionally, the OD value at 450 nm was measured. Six replications were used for each experiment. Cell viability (%) is equal to 100% × (OD sample − OD blank)/(OD control − OD blank).

### 4.5. Anti-Inflammatory and Antioxidant Activity

On a 96-well plate, PC12 cells (5 × 10^4^ cells/well) were planted, cultivated to a density of 70–80%, and the medium was then discarded. Eight groups of cells were created: the control group, the model group, the NMDP group, and three groups of PPT (6.25, 12.5, and 25 M). The comparable group was pre-treated for 2 h with NMDP and various dosages of PPT. The Oxygen-Glucose Deprivation Model/Reperfusion (OGD/R) was subsequently developed.

The LDH, MDA, SOD, TNF-α, and IL-6 contents of the supernatants from each group were then determined using commercial assay kits in accordance with the manufacturer’s instructions.

### 4.6. Animals and Groups

SPF-grade SD rats (male, 250 ± 10 g) were acquired from Changchun Yisi Laboratory Animal Technology Co., Ltd. [Certificate No. SCXK(JI)-20200002]. The rats were provided with standard food and water. In addition, all rats were kept in animal rooms with a room temperature of 21–23 °C, a humidity of 40–60%, and dark-light circulation for 12 h. The Institutional Animal Care and Use Committee of the Jilin University School of Pharmaceutical Science approved protocols for using animals in research. The Ethical Principles for the Use and Care of Animals were followed during the conduct of this investigation.

After a one-week acclimation period, random selection was used to divide SD rats into six groups, with eighteen rats in each group, which was designated as the sham group, model group, NMDP (15 mg/kg) as positive control group, H-PPT (20 mg/kg), M-PPT (10 mg/kg), and L-PPT (5 mg/kg) pre-treatment group. Before building the model, pre-treatment group was intragastric administered once daily for two weeks. On the fourteenth day, an hour after intragastric administration, all groups aside from the sham group had the cerebral ischemia-reperfusion model constructed. According on the outcomes of the preceding pre-experiment, the dose for the PPT treatment group was determined. In addition, the sham and the model groups received an equivalent dose of distilled water. The intragastric dosage was 10 mL/kg of body weight.

### 4.7. Middle Cerebral Artery Occlusion/Reperfusion (MCAO/R)

The intraperitoneal dose of 10% chloral hydrate (350 mg/kg) was used to anesthetize rats. Following Longa’s methodology, the cerebral ischemia-reperfusion damage was established [47]. In a nutshell, the external carotid artery was ligated, and a thread containing silicone at the head end was used to embolize the common carotid artery through the internal carotid artery to the middle cerebral artery. The thread was removed to ensure the formation of reperfusion two hours after the induction of ischemia. The sham group of rats performed an identical procedure without inserting the monofilament.

### 4.8. Neurological Function and Infarct Volume Evaluation

After 2 h ischemia and 22 h reperfusion, the behavioral scores of the rats were assessed using a 0–4 points system by Longa’s scale as follows [47]: 0 points: no symptoms of neurological deficit; 1 point: flexion and inability to extend the forelimb on the contralateral side of the brain injury; 2 points: circling to the left side; 3 points: falling to the left; 4 points: with no walking and consciousness being depressed. The more serious the animal behavior disorder, the higher the score.

After neurobehavioral evaluation, the rats were rapidly decapitated after anesthesia. The entire brain was first divided into 6 successive, 2 mm-thick coronal pieces. Brain slices were then incubated in a 2% TTC solution for 20 min at 37 °C in the dark. The normal brain tissue was red, and the infarcted portion had a white appearance after incubation. A total infarction volume was calculated by combining the values from all the sections after images were collected, using Image Pro Plus 6.0 software to calculate the infarcted area of each slice. Calculations were made to determine the infarction volume ratio and the proportion of the infarcted region to the overall cross-section area.

### 4.9. Brain Edema Assay

After neurobehavioral evaluation, the rats were rapidly decapitated after anesthesia and the brain tissue was placed on filter paper. The brain tissue was immediately put into a small glass vessel, and the wet weight was accurately weighed with a precision electronic balance. Taken in a 105 °C incubator for 12 h to a constant weight, and the water content of brain tissue was calculated according to the dry and wet weight method. The calculation formula is: brain water content (%) = (wet weight − dry weight)/wet weight × 100%.

### 4.10. Blood-Brain Barrier Integrity Assay

Evans blue (EB) is an azo dye with a high affinity for plasma albumin. EB was injected into the rat body at a concentration of 2% (4 mL/kg) via the tail vein for 2 h. The rats were then anesthetized and cardiac perfusion was performed. The injured side brain tissue was stripped and weighed on an electronic balance, 1 mL of DMSO was added per 100 mg of brain tissue, and homogenized with a homogenizer. After mixing, centrifuge at 10,000 rpm for 30 min at 4 °C, drop 200 µL of supernatant and put it in a microplate reader. The OD value was measured with a wavelength of 620 nm.

### 4.11. H&E and Nissl Staining Experiment

The rat brains were embedded in paraffin, formalin-fixed, then coronally sectioned into 5 m pieces. Hematoxylin was used on the sections for 15 min, followed by eosin for 5 min, for the H&E stain. The sections were stained, then cleaned with distilled water, dried, and mounted. In preparation to use Nissl stain, the sections were stained for 5 min with the staining solution, washed with distilled water, dried, and mounted.

### 4.12. Biochemical Evaluation

Weigh 0.1 g of brain tissue, add the brain tissue to pre-cooled saline at a ratio of 1:9, homogenize, and centrifuge for 10 min (3500 rpm). The serum and brain tissue levels of cytokines (TNF-α, IL-1β, IL-6, MDA, and SOD) and serum of NSE levels were determined using a commercial ELISA kit according to the manufacturer’s instructions.

### 4.13. Metabolomics Sample Preparation

A mixture of 100 µL of serum and 300 µL 80% pre-chilled methanol was vortexed, then the mixture was centrifuged at 13,000 rpm for 10 min at 4 °C. The resulting supernatant was then dried by freezing. The dried accumulation was then dissolved in 100 L of 80% methanol and filtered via a syringe channel (0.22 m) to obtain a sample of serum. In order to create the serum QC test for method validation, 10 µL of each test sample were blended.

Then, 0.1 g of brain tissue was taken and homogenized with 80% methanol (1000 μL). The brain test solution was prepared according to the previously mentioned method for serum test solution.

### 4.14. UPLC-Q/TOF-MS Detection Condition

The sample testing was carried out using a Waters Xevo G2-S Q-TOF mass spectrometer with Waters ACQUITY UPLC system in both positive and negative modes.

Conditions for the LC system: chromatographic separation was carried out using a Waters Corporation AC-QUITY UPLC BEH C18 (100 mm × 2.1 mm, 1.7 µm) (MA, United States). The mobile phases contained 0.1% formic acid in eluent A (water) and eluent B (acetonitrile), with a flow rate of 0.4 mL/min, a column temperature of 31 °C, and sample temperature of 16 °C. The following elution parameters were used: 0–2 min, 10% B; 2–26 min, 10–90% B; 26–28 min, 90% B; and 28–30 min, 90–10% B. Acetonitrile was utilized as a weak and robust wash solvent, 10% and 90%, respectively.

Conditions for the MS system: the spectrometer Waters Xevo G2-S Q-TOF-MS was equipped with an electrospray ion source (ESI) operating in different regimes. Capillary voltages in positive and negative modes were 2.6 kV and −2.2 kV, respectively; other operating conditions were as follows: Cone voltage, 40 V; desolvation temperature, 400 °C; cone gas flow, 50 L/h; source temperature, 150 °C; desolvation gas flow, 800 L/h. The high energy function was 20–40 V, and the low energy function was 6 V. Mass Lynx data were collected continuously using the centroid. To assess the stability of the device during the sample test, the QC samples were simultaneously tested four times at random over the whole worklist.

### 4.15. Validation of UPLC-QTOF-MS

The following details the process of validation of the applied method in ESI^+^ and ESI^−^ modes:

System stability: to check the system’s stability, a serum QC sample was taken at random 16 ions (from various spectrum areas) and had their accurate m/z-RT (min) pairs confirmed.

Precision: using five consecutive duplicates of the serum QC sample, the precision was successively assessed.

Reproducibility: five parallel replicates of a single serum sample were examined to determine the reproducibility of sample preparation.

Sample stability: after being prepared, a single blood sample was placed in an autosampler and monitored for 0, 4, 8, 10, and 12 h at 4 °C.

In the preceding experiment, the RSDs of PI and RT in ESI^+^ and ESI^−^ modes were calculated.

### 4.16. Data Processing and Multivariate Analysis

The data collected by UHPLC-Q- TOF-MS were imported into Marker Lynx XS V4.1 software for peaks’ detection, matching, normalization, and alignment. The preprocessed data were then put into Simca-P^®^ software (version 14.1, Umetrics, Umea, Sweden) for principal component analysis (PCA) and orthogonal least squares discriminant analysis (OPLS-DA). A permutation test was also conducted to produce a reference distribution with the R^2^/Q^2^ values suggesting statistical significance. Based on the predicted importance of the variables (VIP > 1) and t-test (*p* < 0.05), potential biomarkers were chosen.

The exact mass charge ratio (error < 10 ppm) and secondary information were compared with metabolite databases such as the Human Metabolome Database (HMDB; http://www.hmdb.ca/, accessed on 20 June 2022) and Metlin (http://metlin.scripps.edu/, accessed on 15 June 2022) to identify potential biomarkers. Hierarchical clustering heat maps were then generated. The Encyclopedia of Genes and Genomes (http://www.kegg.com/, accessed on 25 June 2022) database and MetaboAnalyst (http://www.metaboanalyst.ca/, accessed on 25 June 2022) were used to conduct a metabolic pathway analysis and create maps of the metabolic pathways.

### 4.17. Statistical Analysis

All results data were described as mean values ± standard deviation (SD), the statistical results were conducted using a two-tailed unpaired Student’s t-test or a one-way analysis of variance (ANOVA), and *p* value < 0.05 was regarded as statistically significant.

## 5. Conclusions

This study clarifies the role of PPT in neuroprotection treatment. The ameliorative benefits of PPT on the Oxygen-Glucose Deprivation (OGD)-PC12 cell model in vitro and the cerebral ischemia/reperfusion (CIR) injured rats in vivo were first shown by a systematic pharmacodynamic evaluation. Non-targeted metabolic analysis identified 19 biomarkers responsible for CIR that were controlled by PPT, which was closely related to the disruption of several metabolic pathways, including amino acid metabolism, arachidonic acid metabolism, sphingolipid metabolism, and glycerophospholipid metabolism. The results contributed to understanding the pathogenesis of CIR, discovering the targets for clinical diagnosis, and providing further evidence for using PPT as a neuroprotection agent.

## Figures and Tables

**Table 1:** The endogenous metabolites identified in serum and brain which can be regulated by H-PPT.

NO.	Rt (min)	Metabolite	Formula	*m*/*z*	Δm (PPM)	Adducts	HMDB ID	KEGG ID	Source	Trend	
										M VS. C	H-PPT vs. M
1 *	1.04	L-Phenylalanine	C9H11NO2	166.0870	4.21	[M+H] ^+^	HMDB0000159	C00079	Serum ESI^+^	↑	↓
2a	1.52	2-Aminonaphthalene	C10H9N	144.0815	4.86	[M+H] ^+^	HMDB0041802	C02227	Brain ESI^+^	↑	↓
3a	15.45	Ursodeoxycholic acid	C24H40O4	391.2872	4.60	[M−H] ^−^	HMDB0000946	C07880	Serum ESI^−^	↑	↓
4a	15.48	Hyodeoxycholic acid	C24H40O4	437.2912	0.69	[M+FA−H] ^−^	HMDB0000733	C15517	Brain ESI^−^	↓	↑
5a	15.49	Sphingosine 1-phosphate	C18H38NO5P	378.2435	5.29	[M−H] ^−^	HMDB0000277	C06124	Serum ESI^−^	↑	↓
6a	16.72	LysoPC(18:2(9Z,12Z)/0:0)	C26H50NO7P	520.3389	−1.73	[M+H] ^+^	HMDB0010386	C04230	Serum ESI^+^	↑	↓
7a	17.08	LysoPC(22:6(4Z,7Z,10Z,13Z,16Z,19Z))	C30H50NO7P	568.3411	−1.76	[M+H] ^+^	HMDB0010404	C04230	Serum ESI^+^	↑	↓
8a	17.13	Phosphorylcholine	C5H15NO4P	184.0744	5.98	[M+H] ^+^	HMDB0001565	C00588	Serum ESI^+^	↑	↓
9a	17.63	LysoPC (16:0/0:0)	C24H50NO7P	496.3393	1.01	[M+H] ^+^	HMDB0010382	C04230	Serum ESI^+^	↓	↑
10a	17.7	LysoPC(20:2(11Z,14Z))	C28H54NO7P	570.3544	−1.75	[M+ Na] ^+^	HMDB0010392	C04230	Serum ESI^+^	↑	↓
11a	17.85	6-Hydroxyhexanoic acid	C6H12O3	133.0868	6.76	[M+H] ^+^	HMDB0012843	C06103	Serum ESI^+^	↓	↑
12a	18.69	LysoPC(18:1(9Z)/0:0)	C26H52NO7P	522.3565	2.11	[M+H] ^+^	HMDB0002815	C04230	Serum ESI^+^	↓	↑
13a	19.44	LysoPC(17:0)	C25H52NO7P	510.3564	1.96	[M+H] ^+^	HMDB0012108	C04230	Serum ESI^+^	↓	↑
14a	20.67	LysoPC(15:0/0:0)	C23H48NO7P	480.3075	−4.37	[M−H] ^−^	HMDB0010381	C04230	Brain ESI^−^	↑	↓
15a	20.77	LysoPC(18:0)	C26H54NO7P	524.3726	2.86	[M+H] ^+^	HMDB0010384	C04230	Serum ESI^+^	↓	↑
16a	21.16	Sphingosine	C18H37NO2	344.2818	3.49	[M+FA−H] ^−^	HMDB0000252	C00319	Brain ESI^−^	↑	↓
17a	21.18	3-Dehydrosphinganine	C18H37NO2	300.2887	−3.33	[M+H] ^+^	HMDB0001480	C02934	Brain ESI^+^	↑	↓
18 *	22.24	Vitamin D3	C27H44O	385.3457	−2.08	[M+H] ^+^	HMDB0000876	C05443	Brain ESI^+^	↓	↑
19 *	22.96	Arachidonic acid	C20H32O2	349.2405	6.01	[M+FA−H] ^−^	HMDB0001043	C00219	Brain ESI^−^	↑	↓

*, metabolites validated with standards; a, metabolites confirmed by MS/MS fragments; “C” represents sham group; “M” represents model group.

## Data Availability

Not applicable.

## Supplementary Materials

The supporting information can be downloaded at: https://www.mdpi.com/article/10.3390/ijms24021789/s1.

## Abbreviations

ANOVA	One-way analysis of variance
BPI	Base peak intensity
CIR	cerebral ischemia/reperfusion
CCK-8	Cell-Counting-Kit-8
DMSO	Dimethyl sulfoxide
EB	Evans blue
ELISA	Enzyme-linked immunosorbent assay
ESI	Electrospray ionization
H&E	Hematoxylin and eosin stain
H-PPT	High dosage of PTS
IL-6	Interleukin-6
IL-1β	Interleukin-1β
L-PPT	low dosage of PTS
LDH	Lactate dehydrogenase
MDA	Malondialdehyde
M-PPT	Middle dosage of PTS
NSE	Neuron specific enolase
NMDP	Nimodipine
OGD	Oxygen-glucose deprivation
OPLS-DA	Orthogonal projections to latent structures discriminant analysis
PC12	Rat adrenal pheochromocytoma cells
PCA	Principle component analysis
PI	Peak intensities
PPT	Protopanaxatriol
PBS	Phosphate buffer saline
QC	Quality control
QTOF-MS	Quadrupole time-of-flight mass spectrometry
RSD	Relative Standard Deviation
RT	Retention Time
SD	Standard deviation
SOD	Superoxide dismutase
TNF-α	Tumor necrosis factor-α
TTC	2,3,5-Triphenyl-2H-tetrazolium chloride
UPLC	Ultra-high-performance liquid chromatography
VIP	Variable importance in the projection
