# Supplementary material for: Neuroprotective Effects and Metabolomics Study of Protopanaxatriol (PPT) on Cerebral Ischemia/Reperfusion Injury In Vitro and In Vivo
Source: Int J Mol Sci. 2023 Jan 16;24(2):1789. doi: 10.3390/ijms24021789 (PMC9861888; doi:10.3390/ijms24021789)
Supplement: Supplementary file 1 [file ijms-24-01789-s001.zip › ijms-2116002-supplementary.pdf]

Electronic supplementary information

Table S1. The RSDs (%) of PI and RT in validation tests

Tests	ESI^+ mode		ESI^- mode	
	PI	RT	PI	RT
system stability	1.81~4.73	0.31~1.24	1.60~4.62	0.25~1.80
precision	0.83~3.36	0.14~0.28	1.65~3.31	0.18~0.55
reproducibility	1.25~3.57	0.16~1.49	0.65~3.68	0.06~0.65
Sample stablilty	1.36~3.24	0.08~0.46	1.96~3.25	0.14~0.87

The abovementioned validation showed that the UPLC-QTOF-MS method was effective in terms of precision, repeatability, and stability.

Table S2. The consequences from metabolic pathways

Pathway Name	Match Status	p	-log (p)	Holm p	FDR	Impact
Phenylalanine, tyrosine and tryptophan biosynthesis	1/4	0.0206	1.6881	1	0.5742	0.50
Phenylalanine metabolism	1/10	0.0516	1.2961	1	1	0.36
Arachidonic acid metabolism	1/36	0.1858	0.7651	1	1	0.31
Sphingolipid metabolism	3/21	0.1084	3.9390	0.0097	0.0097	0.14
Glycerophospholipid metabolism	9/36	0.1858	1.8712	1	0.56506	0.03

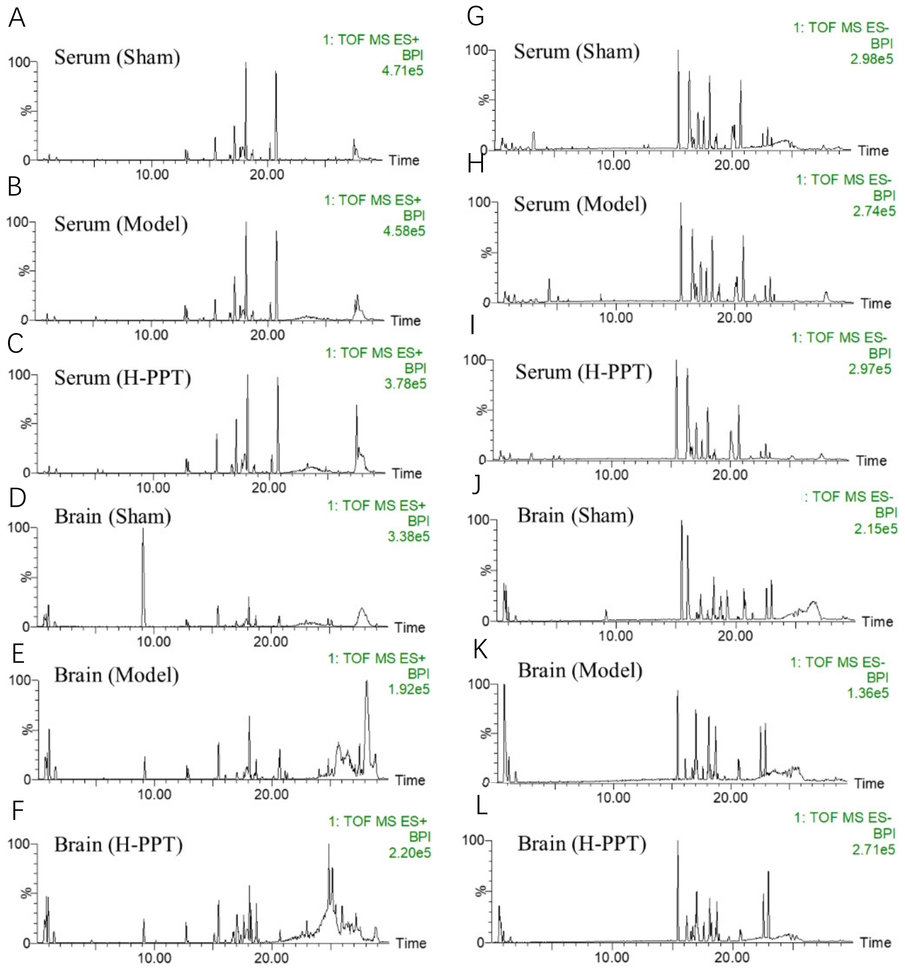

Figure S1. The serum and brain samples from the Sham, Model, and H-PPT groups are shown in the representative BPI chromatograms in positive (A-F) and negative (G-L) modes.
